# Lung ultrasound-guided treatment for heart failure: An updated meta-analysis and trial sequential analysis

**DOI:** 10.3389/fcvm.2022.943633

**Published:** 2022-08-22

**Authors:** Yan Li, Hu Ai, Na Ma, Peng Li, Junhong Ren

**Affiliations:** ^1^Department of Ultrasound Medicine, Beijing Hospital, National Center of Gerontology, Institute of Geriatric Medicine, Chinese Academy of Medical Sciences, Beijing, China; ^2^Deparment of Cardiology, Beijing Hospital, National Center of Gerontology, Institute of Geriatric Medicine, Chinese Academy of Medical Sciences, Beijing, China; ^3^The Key Laboratory of Geriatrics, Beijing Institute of Geriatrics, Institute of Geriatric Medicine, Chinese Academy of Medical Sciences, Beijing Hospital, National Center of Gerontology, National Health Commission, Beijing, China

**Keywords:** lung ultrasound, heart failure, adverse cardiac events, prognosis, meta-analysis

## Abstract

**Background:**

The usefulness of lung ultrasound (LUS) in guiding heart failure (HF) treatment is still controversial.

**Purpose:**

We aimed to evaluate the usefulness of LUS-guided treatment vs. usual care in reducing the major adverse cardiac event (MACE) rate in patients with HF.

**Materials and methods:**

We performed a systematic review and meta-analysis of randomized controlled trials (RCTs) identified through systematic searches of MEDLINE, EMBASE, the Cochrane Database, Google Scholar, and SinoMed. The primary outcome was MACEs (a composite of all-cause mortality, HF-related rehospitalization, and symptomatic HF). The required information size was calculated by trial sequential analysis (TSA).

**Results:**

In total, ten RCTs involving 1,203 patients were included. Overall, after a mean follow-up period of 4.7 months, LUS-guided treatment was associated with a significantly lower risk of MACEs than usual care [relative risk (RR), 0.59; 95% confidence interval (CI), 0.48–0.71]. Moreover, the rate of HF-related rehospitalization (RR, 0.63; 95% CI, 0.40–0.99) and N-terminal pro-B-type natriuretic peptide (NT-proBNP) concentration (standardized mean difference, –2.28; 95% CI, –4.34 to –0.22) were markedly lower in the LUS-guided treatment group. The meta-regression analysis showed a significant correlation between MACEs and the change in B-line count (*p* < 0.05). The subgroup analysis revealed that the risk of MACEs was markedly lower in patients aged up to 70 years (RR, 0.54; 95% CI, 0.44–0.67), with a lower rate of atrial fibrillation (< 27.2%) (RR, 0.53; 95% CI, 0.43–0.67), and with a lower NT-proBNP concentration (< 3,433 pg/ml) (RR, 0.51; 95% CI, 0.40–0.64). TSA indicated a lower risk of MACEs with LUS-guided treatment than with usual care among patients with HF (*p* < 0.05).

**Conclusion:**

Lung ultrasound seems to be a safe and effective method to guide HF treatment.

**Systematic review registration:**

[https://inplasy.com/], identifier [INPLASY202220124].

## Introduction

Lung ultrasound (LUS) is a dynamic, convenient, repeatable, non-radioactive, and semi-conductive method that can be used to evaluate extravascular lung water (ELWI) (1). B-lines, which are an imaging manifestation on LUS, are closely related to the severity of ELWI (2, 3). Pulmonary circulation congestion is a common pathophysiological manifestation in patients with heart failure (HF) (4). In clinical practice, New York Heart Association (NYHA) functional class or Killip classification, N-terminal pro-B-type natriuretic peptide (NT-proBNP), and chest X-ray are commonly used to evaluate HF severity and prognosis (5). The lungs have long been considered a restricted area for ultrasound examination because gases in the lungs block ultrasound beam propagation. However, in the recent years, the interpretation of ultrasound artifacts has increased the use of LUS in emergency critical care medicine, cardiology, and other fields (6, 7). B-lines are vertical reverberation artifacts that appear at the pleural line and extend to the bottom of the screen. Ultrasonography can show a large number of B-lines, which indicate lung interstitial syndrome. The B-line count increases with the decrease in air content and the increase in lung tissue density (8). The B-line count correlates with Kerley B-lines and lung water scores on chest radiography (9) and with ELWI measured by invasive thermodilution (10).

A previous study showed that the B-line count has a similar accuracy to B-type natriuretic peptide concentration in diagnosing HF (11). Other studies have shown that B-lines on LUS can be used for risk stratification in patients with HF and to predict the occurrence of adverse cardiac events (12, 13). In patients with acute HF (AHF), ≥ 15 B-lines on 28-zone LUS at discharge identified patients with a 5-fold increased risk of HF readmission or mortality. Meanwhile, in ambulatory patients with chronic HF (CHF), ≥ 3 B-lines on five- or eight-zone LUS identified patients with a nearly 4-fold increased risk of 6-month HF hospitalization or mortality (14). Therefore, similar to BNP, LUS has clinical value in guiding the management of patients with HF.

However, the role of LUS in guiding HF therapy is still controversial (15–17). Systematic reviews on this topic have thus far been inconclusive, providing conflicting results. In a recent systematic review and meta-analysis, a total of three randomized controlled trials (RCTs) involving 493 patients with HF were included. A total of 251 patients were managed with LUS + physical examination (PE)-guided treatment, whereas 242 subjects were managed with PE-guided therapy alone (18). The mean follow-up period was 5.0 months. This meta-analysis demonstrated that outpatient LUS-guided diuretic therapy reduces urgent visits for worsening HF symptoms [relative risk (RR), 0.32; 95% confidence interval (CI), 0.18–0.59]. However, the rates of HF hospitalization (RR, 0.65; 95% CI, 0.34–1.22) and all-cause mortality (RR, 1.39; 95% CI, 0.68–2.82) were similar between the two groups. However, only three RCTs and 493 patients were included in this meta-analysis, and the study sample was too small to explain the other clinical outcomes. In addition, the study did not perform meta-regression and/or subgroup analyses or analyze other parameters, such as cardiac function, quality of life, and length of hospital stay (19), which should be evaluated in other studies (20).

As the amount of available evidence has recently increased, we performed this updated meta-analysis and trial sequential analysis (TSA) to evaluate the effect of LUS-guided treatment on major adverse cardiac events (MACEs) in patients with HF.

## Materials and methods

### Data source and search strategy

We searched MEDLINE (source, PubMed from 2005 to December 2021), EMBASE (2005 to December 2021), the Cochrane Central Register of Controlled Trials (to December 2021), Google Scholar (to December 2021), SinoMed (to December 2021), and the ClinicalTrials.gov website (to December 2021) using the terms “heart failure,” “lung ultrasound,” “heart failure visits,” “heart failure rehospitalization,” and “randomized trial.” Manual checking of the reference lists of all relevant articles was performed. No restrictions were applied. The review is registered at https://inplasy.com/ (INPLASY202220124).

### Study selection

We first conducted initial screening of titles and abstracts, which was followed by full-text review. Studies were considered eligible if they met the following criteria: (1) included patients with AHF or CHF; (2) included HF patients who underwent LUS-guided treatment plus usual care vs. usual care alone; (3) MACE (HF-related rehospitalization or all-cause mortality) was the primary outcome of interest; and (4) RCT design.

The exclusion criteria were as follows: (1) patients with cardiac shock; (2) patients complicated with pneumonia; (3) single-arm study; (4) no primary outcome; (5) retrospective study, animal study, case report, or review; and (6) duplicated data.

### Data extraction

A number of two reviewers extracted the data on patients’ characteristics, the LUS used, the study quality, and clinical outcomes using a standard data collection form. Disagreements were resolved by discussion.

The primary outcome was the rate of MACEs. The secondary outcomes were the rates of HF-related rehospitalization, all-cause mortality, length of hospital stay, change in NT-proBNP concentration, diuretic dose, quality of life, and rate of adverse events (acute kidney injury and hypokalemia).

### Quality assessment

The Preferred Reporting Items for Systemic reviews and Meta-Analyses statement was followed (21). A number of two reviewers assessed the quality of the selected studies. The components used for quality assessment were the methods used for random sequence generation, allocation concealment, blinding of the outcome assessment, and selective outcome reporting (22).

### Data synthesis and analysis

The results were analyzed quantitatively with STATA 14.0 software (Stata Corp., College Station, CA, United States) using the fixed-effects model. We calculated the pooled RR of dichotomous outcomes and the standardized mean difference (SMD) or weighted mean difference (WMD) for continuous data with 95% CIs.

Heterogeneity was examined using the *I*^2^ statistic and the chi-square test. An *I*^2^ statistic of > 50% was considered to indicate substantial heterogeneity (23). Once heterogeneity was noted, the between-study sources of heterogeneity were investigated using the subgroup analysis by stratifying the original estimates according to the study characteristics. Publication bias was quantitatively assessed using Egger’s regression (*p* ≤ 0.10) (24) and qualitatively assessed by visual inspection of funnel plots of the logarithm of RR vs. the standard error (25).

The univariate meta-regression analysis was used to identify possible contributors to between-study variance. In particular, we investigated the associations between the RRs of MACEs, HF-related rehospitalization, symptomatic HF, and clinically plausible factors, including AHF, patient number, age, atrial fibrillation (AF), diabetes mellitus (DM), ischemic HF, left ventricular ejection fraction (LVEF), troponin I (TnI), estimated glomerular filtration rate (eGFR), B-line count, change in B-line count, NT-proBNP concentration, and follow-up duration. Sensitivity analyses were conducted to determine the influence of individual RCTs on the overall pooled results. All analyses were performed according to the intention-to-treat principle. Statistical significance was set at 0.05 for the *Z*-test for RR.

### Subgroup analysis

Based on the baseline condition of the patients (AHF vs. CHF), the studies were divided into “AHF” and “CHF” subgroups. In addition, based on the mean rates of baseline clinical factors (age, AF, DM, ischemic HF, LVEF, TnI, eGFR, B-line count, NT-proBNP concentration, and follow-up duration), all studies were classified into “age < 70.0 years” and “age ≥ 70.0 years” subgroups, “AF < 27.2%” and “AF ≥ 27.2%” subgroups, “DM < 38.3%” and “DM ≥ 38.3%” subgroups, “ischemic HF < 44.2%” and “ischemic HF ≥ 44.2%” subgroups, “LVEF < 37.5%” and “LVEF ≥ 37.5%” subgroups, “TnI < 1.23 ng/mL” and “TnI ≥ 1.23 ng/mL” subgroups, “eGFR < 48.8 mL/min/1.73 m^2^” and “eGFR ≥ 48.8 mL/min/1.73 m^2^” subgroups, “B-line count < 5.0” and “B-line count ≥ 5.0” subgroups, and “follow-up < 4.7 months” and “follow-up ≥ 4.7 months” subgroups.

### Trial sequential analysis

In this meta-analysis, TSA was used to reduce the risk of reaching a false-negative conclusion (26). When the cumulative Z-curve crossed the trial sequential monitoring boundary or entered the futility area, a sufficient level of evidence for the anticipated intervention effect was reached, and no further trials were needed. If the Z-curve did not cross any of the boundaries and the required information size (RIS) had not been reached, the evidence was deemed insufficient to reach a conclusion, and more trials were needed to confirm the results. In this TSA for MACEs, HF-related rehospitalization, and symptomatic HF, we estimated the RIS based on a RR reduction of 20%. The type I error (α) = 0.05 (two-sided) and power (1−β) = 0.80. The control event proportions were 32% for MACEs, 20% for HF-related rehospitalization, and 32% for symptomatic HF, which were calculated from the comparator group. The *I*^2^ values were 40, 55, and 65% for MACEs, HF-related rehospitalization, and symptomatic HF, respectively. TSA was conducted using TSA software, version 0.9.5.10 Beta.^1^

## Results

### Search results

**FIGURE 1:**
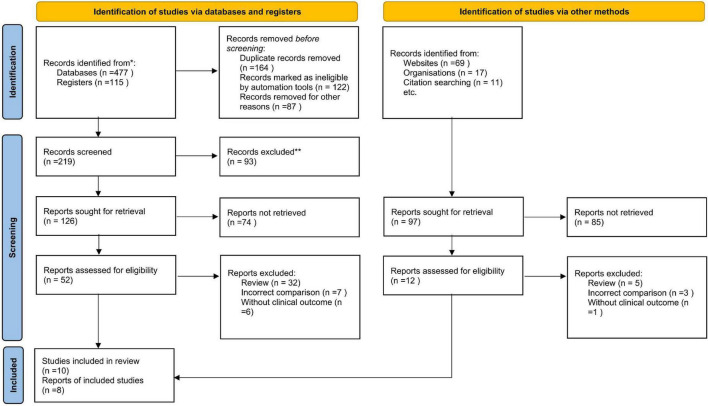
Flowchart of study selection.

We initially identified 357 potentially relevant articles. In total, seventy studies were considered to be of interest and were retrieved for full-text review. In total, sixty articles were excluded owing to duplication (*n* = 6), incorrect study type (reviews) (*n* = 32), incorrect comparisons (*n* = 11), and no clinical outcomes (*n* = 5). Therefore, 10 RCTs were finally included in the analysis. Figure 1 is a flowchart showing the process of study selection.

### Study characteristics

A total of ten published RCTs (15–17, 27–33) involving a total of 1,203 patients were included. Of these studies, eight were completed and reported clinical outcomes (15–17, 27, 29, 30, 32, 33). However, the other two studies were still in the process of subject enrollment (28, 31). A total of three RCTs (27, 30, 31) enrolled patients with AHF, five RCTs (15, 16, 28, 29) enrolled patients with CHF, and one RCT (30) enrolled patients with CHF plus pulmonary artery hypertension. Another RCT (33) enrolled subjects admitted to hospital with cardiopulmonary symptoms, nearly half of whom were diagnosed with AHF. In six RCTs (15–17, 27–29), the diuretic dose was modified according to the results of LUS. Meanwhile, diuretic treatment was given based on LUS and echocardiography (30), body weight (32), and inferior vena cava ultrasound (31, 33). In the usual care group, the diuretic treatment strategy was optimized according to the results of PEs, blood tests, echocardiography, and chest X-ray in all RCTs. The primary endpoint was the rate of MACEs (composite of death, hospital readmission, urgent HF visits, HF-related rehospitalization, and so on) in eight RCTs. However, in the other two RCTs, the rate of patients with fewer B-lines at 6 h after enrollment and the length of hospital stay were the primary outcomes (17, 33; Table 1).

**TABLE 1:** Baseline characteristics of the included randomized controlled trials.

Trial/first author	Study design	Patients	LUS-guided treatment regimen	PE-guided treatment regimen	Primary endpoint
Ding (27)	RCT	AHF	(1) when the number of B-lines was < 11, maintain the current diuretic treatment regimen; (2) when the number of B-lines was 11–20, increase diuretic dose; (3) when the number of B-lines was 21–32, increase larger diuretic dose;	Based on clinical assessment, the diuretic dose will be adjusted according to signs and symptoms of clinical congestion.	Composite event of all-cause mortality, acute kidney injury, cardiac shock, ventricular fibrillation, and ventricular tachycardia with disturbed hemodynamics
LUS-HF (15)	RCT	CHF	Increase the diuretic regime when the number of B-lines across the eight chest zones was more than 3.	The diuretic dose will be adjusted according to the clinical assessment.	Composite event of urgent visits, hospitalization for worsening HF and death from all cause
EPICC (28)	RCT	CHF	Treatment will be optimized and diuretic doses increased in the presence of at least one positive bilateral pulmonary region and/or significant pleural effusion (> 1 cm)	Based on clinical assessment, the diuretic dose will be adjusted according to signs and symptoms of clinical congestion.	Combination of cardiovascular death and readmission for HF at 6 months.
Marini (16)	RCT	CHF	Loop diuretic dose was modified according to LUS B-line’ number	Diuretic treatment was optimized according to PE, blood tests, echocardiogram, and chest X-ray.	Rates of hospitalization for acute decompensated HF at 90°d follow-up
CLUSTER-HF (29)	RCT	CHF	A pre-specified diuretic dosage protocol (at least 80–120 mg PO of furosemide equivalent/day) was suggested when the number of B-lines was ≥ 3.	Diuretic treatment was optimized according to PE, blood tests, echocardiogram, and chest X-ray.	Composite event of urgent HF visits, rehospitalization for worsening HF and all-cause mortality
Li (30)	RCT	CHF + PAH	Diuretic dose was modified according to the results of LUS and echocardiography.	Diuretic treatment was optimized according to PE, blood tests, echocardiogram, and chest X-ray.	Rate of adverse event, including respiratory failure, pulmonary embolism, and stroke
Risk-HF (31)	RCT	AHF	Risk-guided intervention, including (1) fluid management guided with lungs and inferior vena cava ultrasound, (2) post-discharge follow-up, (3) optimal drug titration, (4) better transition of care, (5) intensive self-care education, (6) exercise guidance.	Standard post-discharge hospital care	Rate of death and/or hospital readmissions at 30°d post-discharge
BLUSHED-AHF (17)	RCT	AHF	Diuretic treatment was given (1 × oral diuretic dose, or repeat, or double original IV diuretic dose), until there is a decrease in B-lines to ≤ 15, 6 h of care has been delivered, or the patient has been discharged.	Diuretic treatment was optimized according to PE, blood tests, echocardiogram, and chest X-ray.	Rate of patients ≤ 15 B-lines at 6°h after enrollment
Huang (32)	RCT	CHF	Diuretic dose was modified according to the results of LUS and body weight.	Diuretic treatment was optimized according to PE, blood tests, echocardiogram and chest X-ray.	Rate of hospital readmissions at 90°d post-discharge
IMFCU-1 (33)	RCT	Patients admitted with cardiopulmonary symptoms	Treatment was guided by the focused clinical ultrasonography of the heart, lung, and lower extremity veins	Clinical decisions were based on clinical evaLUSation and other further investigations.	Mean length of hospital stay

RCT, randomized controlled trial; AHF, acute heart failure; CHF, chronic heart failure; PAH, pulmonary artery hypertension; LUS, lung ultrasound; PE, physical examination.

Among the 1,203 patients enrolled patients, the average age was . The total number of patients in each study ranged from 44 to 68, and the follow-up duration was 69.9 years. Approximately 27.2% of patients had AF, 38.3% had DM, and 44.2% had ischemic HF. The average LVEF was 37.5%, TnI concentration was 1.23 ng/ml, eGFR was 48.8 ml/min/1.73 m^2^, B-line count was 5, and NT-proBNP concentration was 3,433 pg/ml. Most patients were followed up for < 6 months, and the average follow-up period was 4.7 months (Table 2).

**TABLE 2:** Baseline characteristics of patients in the lung ultrasound-guided treatment and usual care groups.

First author	Year	Patient’s num.	Age, year	AF, %	DM, %	IHF, %	LVEF, %	TnI, ng/ml	eGFR, ml/min	B line’ num.	Change of B line num.	NT-proBNP, pg/ml	Fol., m
Ding et al. (27)	2018	113/112	67.3/68.5	10.6/12.5	33.6/30.4	25.7/23.2	46.7/48.4	0.6/0.7	58.4/64.8	NR	NR	2374.6/1922.6	13
LUS-HF (15)	2019	61/62	69.0/69.0	61.0/49.0	44.0/38.0	31.0/38.0	39.0/39.0	NR	62.0/65.0	4.0/4.0	−1.0/−0.4	1897.0/1559.0	6
EPICC (28)	2019	76/76	≥ 18	NR	NR	NR	< 50.0	NR	<3&	NR	NR	> 1000.0	6
Marini (16)	2020	127/117	73.2/69.8	21.2/23.1	28.3/35.9	87.0/79.0	32.2/30.7	NR	1.4/1.4&	1.4/NR	−0.5/NR	1559.0/1319.0	3
CLUSTER-HF (29)	2020	63/63	62.0/63.0	15.8/12.3	38.1/38.4	61.9/56.9	30.0/34.9	0.12/0.09	26.9/23.0*	1.0/1.5	−8.3%/−5.8%§	4067.0/5183.0	6
Li (30)	2020	50/50	67.0/67.3	NR	NR	36.0/38.0	35.6/36.2	0.6/0.6	1.6/1.6@	21.0/20.2	−16.4/−10.0	3896.8/3930.5$	3
Risk-HF (31)	2020	202/202	≥ 18	NR	NR	NR	NR	NR	NR	NR	NR	NR	3
BLUSHED-AHF (17)	2021	66/64	66.0/64.0	NR	47.0/43.8	NR	41.3/38.8	3.7/3.6	1.4/1.4&	47.5/49.7	−25.3/−19.7	6810.5/7814.8	3
Huang (32)	2021	50/50	74.6/74.9	NR	NR	NR	NR	NR	NR	NR	NR	NR	3
IMFCU-1 (33)	2021	124/124	80.1/79.0	NR	32.3/37.1	23.4/18.5	NR	NR	NR	9.2/6.4	−4.3/4.9	NR	1

#, Killip class; $, BNP; &, serum creatinine; *, rates of chronic kidney disease (eGFR 16–60 mL/min/1.73 m^2^); @, Cystatin C; §, change in the percentage of patients with ≥ 3 B-lines.

LUS, lung ultrasound; Num., number; AF, atrial fibrillation; DM, diabetes mellitus; IHF, ischemic heart failure; LVEF, left ventricular ejection fraction; TnI, troponin I; NYHA, New York Heart Association; NT-proBNP, N-terminal pro-B-type natriuretic peptide; Fol., follow; m., month; NR, not reported.

### Methodological quality assessment

A total of ten RCTs randomized the participants. However, one RCT did not report the methodological details of random sequence generation (30). A total of six studies used satisfactory methods of treatment allocation concealment (15–17, 28–31). Blinding of participants and personnel was reported in five studies (15–17, 28, 29). There was a low risk of attrition bias and reporting bias in all studies.

### Primary endpoint

**FIGURE 2:**
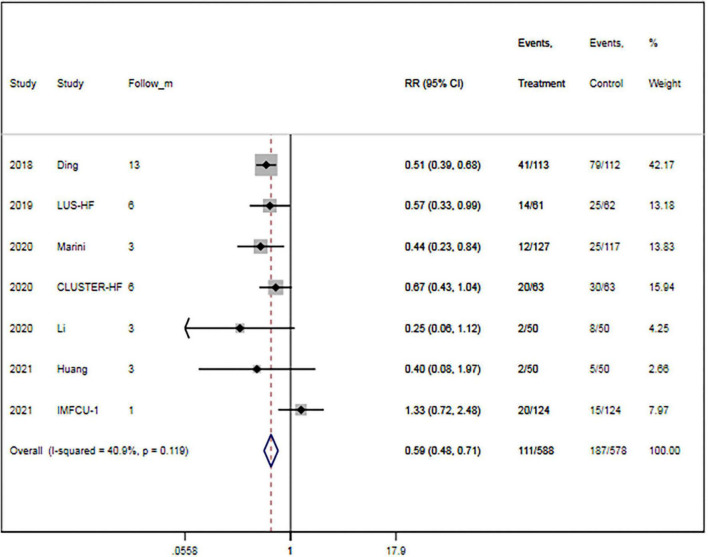
Lung ultrasound-guided treatment is associated with a decreased risk of major adverse cardiac event. Fixed-effects model (*I*^2^ = 40.9%). LUS, lung ultrasound; MACE, major adverse cardiac event; RR, relative risk; CI, confidence interval.

A total of seven trials (15, 16, 27, 29, 30, 32, 33) provided data on MACEs. There were 588 patients in the LUS-guided treatment group and 578 patients in the usual care group. Compared to the usual care group, the LUS-guided treatment group was associated with a significantly lower risk of MACEs (RR, 0.59; 95% CI, 0.48–0.71; *p* < 0.001) (Figure 2). There was a low level of heterogeneity (*I*^2^ = 40.9%). The funnel plot did not show asymmetry in Begg’s test (*p* = 0.652) or Egger’s test (*p* = 0.359). Moreover, a sensitivity analysis was performed by removing each of the trials one at a time, which did not have any influence on the rate of MACEs (Table 3).

**TABLE 3:** Primary and secondary outcomes of the lung ultrasound-guided treatment group and the usual care group.

Outcomes	Num. of event in LUS group	Num. of event in usual care group	RR/SMD/WMD (95% CI)	*P*	*I* ^2^
**Primary outcome**					
MACE	111/588	187/578	0.59 (0.48 to 0.71)β	< 0.001	40.9
**Secondary outcomes**					
All-cause mortality	28/414	26/404	1.06 (0.64 to 1.75)β	0.825	0
HF related rehospitalization	75/588	118/578	0.63 (0.40 to 0.99)β	0.046	54.7
Rate of less B-line number	141/190	128/189	1.09 (0.96 to 1.24)β	0.169	0
Hypokalemia	8/124	11/125	0.73 (0.30 to 1.76)β	0.487	40.0
Acute kidney injury	8/118	11/125	0.78 (0.12 to 5.15)β	0.793	71.7
Changes of B-lines	932	920	−3.86 (−8.09 to 0.38)§	0.169	97.3
Quality of life	932	920	1.55 (−0.14 to 3.24)ζ	0.073	98.4
Diuretic dosage	932	920	−0.88 (−0.21 to 1.97)ζ	0.113	98.1
Duration of hospitalization	932	920	−1.56 (−3.36 to 0.24)§	0.090	99.0
NT-proBNP level	932	920	−2.28 (−4.34 to −0.22)ζ	0.030	99.0

β, RR, relative risk; ζ, SMD, standardized mean difference; §, WMD, weighted mean difference.

MACE, major adverse cardiac event; HF, heart failure; NT-proBNP, N-terminal pro-B-type natriuretic peptide; LUS, lung ultrasound.

### Secondary endpoints

**FIGURE 3:**
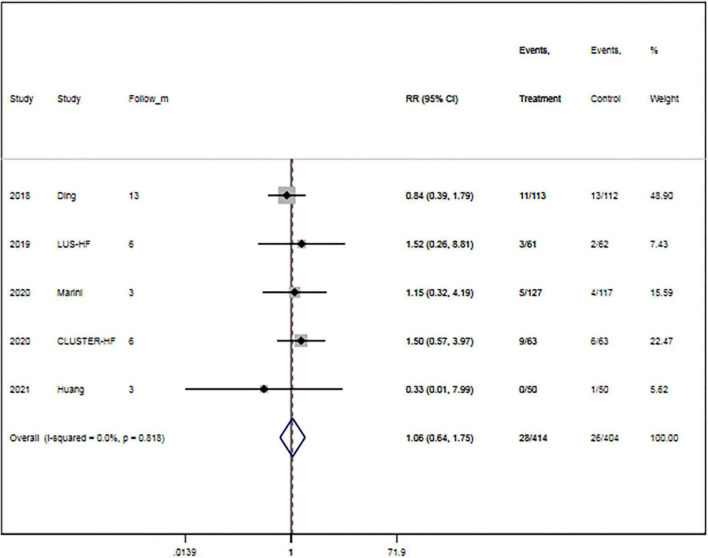
Lung ultrasound-guided treatment is not associated with a decreased risk of mortality. Fixed-effects model (*I*^2^ = 0). LUS, lung ultrasound; RR, relative risk; CI, confidence interval.

The rate of all-cause mortality was specified in five trials (15, 16, 27, 29, 32). Overall, LUS-guided treatment was associated with a comparable rate of all-cause mortality to usual care (RR, 1.06; 95% CI, 0.64–1.75; *p* = 0.825) (Figure 3). There was a low level of heterogeneity (*I*^2^ = 0.0%).

**FIGURE 4:**
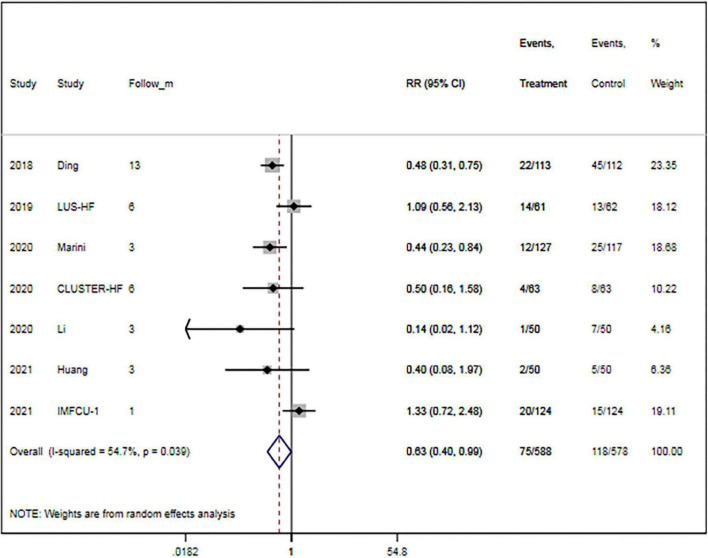
Lung ultrasound-guided treatment is associated with a decreased risk of heart failure-related rehospitalization. Random-effects model (*I*^2^ = 54.7%). LUS, lung ultrasound; HF, heart failure; RR, relative risk; CI, confidence interval.

The rate of rehospitalization for worsening HF was specified in seven trials (15, 16, 27, 29, 30, 32, 33). Overall, LUS-guided therapy was associated with a significantly lower rate of rehospitalization for worsening HF than usual care (RR, 0.63; 95% CI, 0.40–0.99; *p* = 0.046) (Figure 4). Although there was a high level of heterogeneity (*I*^2^ = 54.7%), the funnel plot did not show marked asymmetry in Begg’s test (*p* = 0.652) or Egger’s test (*p* = 0.812).

A total of three studies (15, 29, 31) reported the rate of patients with fewer B-lines. Overall, LUS-guided treatment was associated with a similar rate of patients with fewer B-lines (RR, 1.09; 95% CI, 0.96–1.24; *p* = 0.169) as usual care.

**FIGURE 5:**
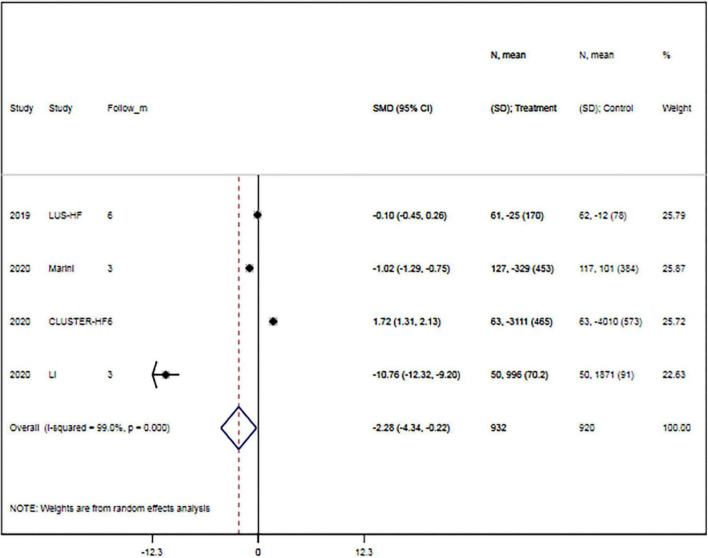
Lung ultrasound-guided treatment is associated with a lower N-terminal pro-B-type natriuretic peptide concentration. Random-effects model (*I*^2^ = 99.0%). LUS, lung ultrasound; NT-proBNP, N-terminal pro-B-type natriuretic peptide; SMD, standardized mean difference; CI, confidence interval.

The rates of hypokalemia (RR, 0.73; 95% CI, 0.30–1.76; *p* = 0.487) and acute kidney injury (RR, 0.78; 95% CI, 0.12–5.15; *p* = 0.793) were comparable between groups. Meanwhile, changes in B-line count (WMD, –3.86; 95% CI, –8.09 to 0.38; *p* = 0.169), quality of life (SMD, 1.55; 95% CI, –0.14 to 3.24; *p* = 0.073), diuretic dose (SMD, –0.88; 95% CI, –0.21 to 1.97; *p* = 0.113), and hospitalization duration (WMD, –1.56; 95% CI, –3.36 to 0.24; *p* = 0.090) were also comparable between the two groups. However, LUS-guided treatment was associated with a significantly lower NT-proBNP concentration than usual care (SMD, –2.28; 95% CI, –4.34 to –0.22; *p* = 0.030) (Figure 5 and Table 3).

### Meta-regression analysis

**FIGURE 6:**
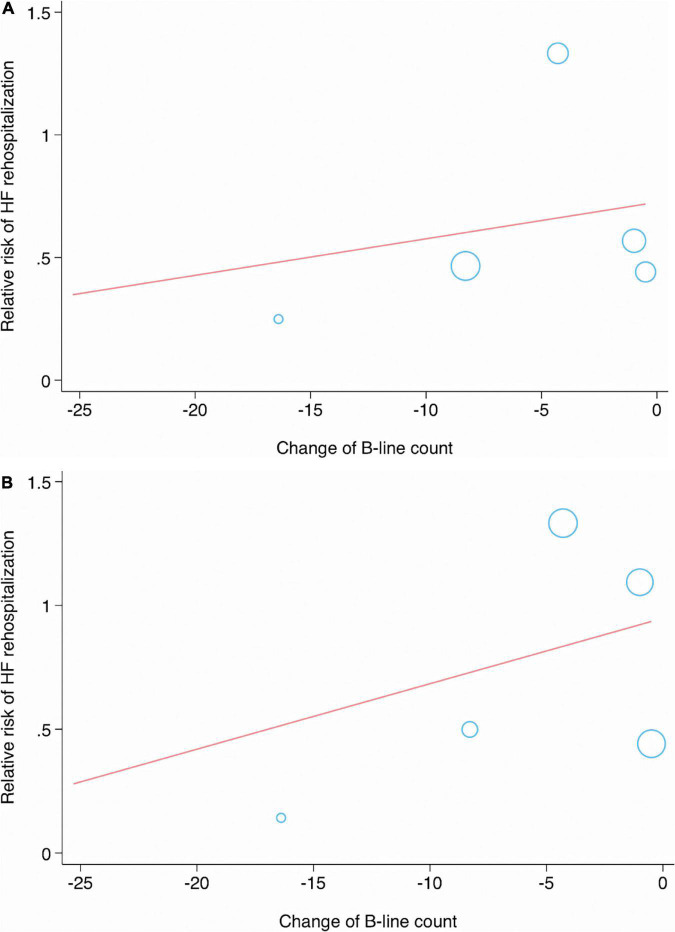
Meta-regression analysis showing significant associations between change in B-line count and major adverse cardiac event **(A)**, or HF rehospitalization **(B)**.

In the meta-regression analysis, the condition of patients (*p* = 0.434), sample size (*p* = 0.454), age (*p* = 0.222), AF (*p* = 0.928), DM (*p* = 0.544), ischemic HF (*p* = 0.473), LVEF (*p* = 0.746), TnI (*p* = 0.653), eGFR (*p* = 0.980), B-line count (*p* = 0.723), change in B-line count (*p* = 0.033) (Figure 6A), NT-proBNP concentration (*p* = 0.625), and follow-up duration (*p* = 0.252) were not significantly associated with the pooled RR of MACEs.

No significant correlations were observed between the RR of HF-related rehospitalization and the condition of patients (*p* = 0.688), sample size (*p* = 0.898), age (*p* = 0.110), AF (*p* = 0.259), DM (*p* = 0.303), ischemic HF (*p* = 0.380), LVEF (*p* = 0.965), TnI (*p* = 0.989), eGFR (*p* = 0.491), B-line count (*p* = 0.616), change in B-line count (*p* = 0.039) (Figure 6B), NT-proBNP concentration (*p* = 0.675), and follow-up duration (*p* = 0.344) (Table 4).

**TABLE 4:** Meta-regression analysis of baseline data and clinical outcomes.

Factors	MACE			HF related rehospitalization		
	β-coefficient	95% CI	*P*	β-coefficient	95% CI	*P*
AHF	−0.253	−1.021 to 0.513	0.434	−0.190	−1.409 to 1.030	0.688
Patient’s num	0.002	−0.004 to 0.008	0.454	0.001	−0.011 to 0.012	0.898
Mean age (yr)	0.027	−0.023 to 0.079	0.222	0.065	−0.022 to 0.153	0.110
AF	0.001	−0.025 to 0.027	0.928	0.012	−0.022 to 0.047	0.259
DM	0.030	−0.111 to 0.171	0.544	0.069	−0.107 to 0.245	0.303
Ischemic HF	−0.005	−0.025 to 0.139	0.473	−0.008	−0.030 to 0.014	0.380
Mean LVEF	−0.005	−0.051 to 0.041	0.746	0.001	−0.096 to 0.099	0.965
Mean TnI	−0.335	−7.351 to 6.681	0.653	−0.018	−13.076 to 13.079	0.989
Mean eGFR	0.0001	−0.016 to 0.016	0.980	0.005	−0.017 to 0.028	0.491
Mean B-line count	−0.004	−0.004 to 0.028	0.723	−0.010	−0.065 to 0.045	0.616
Change of B-line count	−0.015	−0.0004 to −0.722	0.033	−0.029	−0.001 to −0.841	0.039
Mean NT-proBNP	0.0001	−0.0002 to 0.0002	0.625	−0.0001	−0.0009 to 0.006	0.675
Mean follow-up	−0.027	−0.082 to 0.027	0.252	−0.042	−0.152 to 0.068	0.344

MACE, major adverse cardiac event; AHF, acute heart failure; CHF, chronic heart failure; LVEF, left ventricular ejection fraction; NT-proBNP, N-terminal pro-B-type natriuretic peptide; HF, heart failure; Num., number; RR, relative risk.

### Subgroup analysis

**FIGURE 7:**
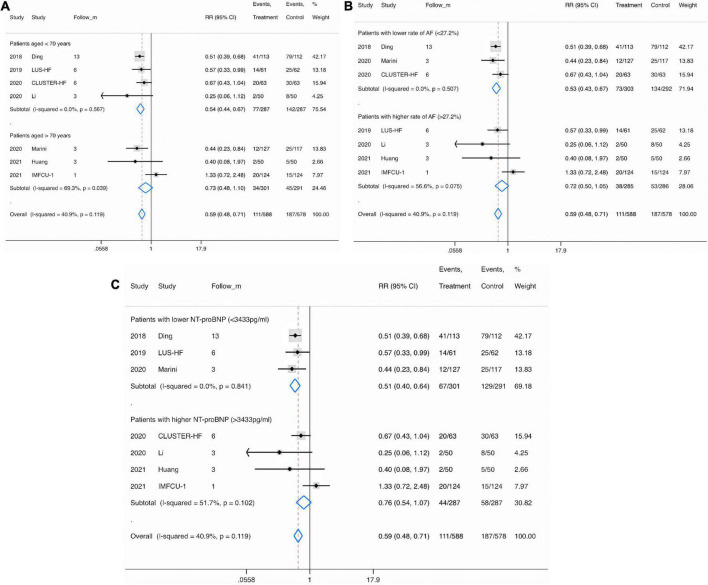
Subgroup analysis for major adverse cardiac event in patients specified by age **(A)**, atrial fibrillation **(B)**, and N-terminal pro-B-type natriuretic peptide concentration **(C)**. Fixed-effects model (*I*^2^ = 40.9%). LUS, lung ultrasound; MACE, major adverse cardiac event; RR, relative risk; CI, confidence interval.

In the subgroup analysis, the pooled RRs of MACEs in studies involving patients aged up 70 years (RR, 0.54; 95% CI, 0.44−0.67), with a lower rate of AF (< 27.2%) (RR, 0.53; 95% CI, 0.43−0.67), or with a lower NT-proBNP concentration (< 3,433 pg/ml) (RR, 0.51; 95% CI, 0.40−0.64) were significantly lower than in studies involving patients aged ≥ 70 years (RR, 0.73; 95% CI, 0.48−1.10) (Figure 7A), with a higher rate of AF (≥ 27.2%) (RR, 0.72; 95% CI, 0.50−1.05) (Figure 7B), or with a higher NT-proBNP concentration (≥ 3,433°pg/ml) (RR, 0.76; 95% CI, 0.64−1.07) (Figure 7C).

Meanwhile, the pooled RRs of HF-related rehospitalization in studies enrolling patients aged up to 70 years (RR, 0.56; 95% CI, 0.40−0.79), with a lower rate of AF (< 27.2%) (RR, 0.47; 95% CI, 0.33−0.67), DM (< 38.3%) (RR, 0.61; 95% CI, 0.45−0.82), or with lower LVEF (< 37.5%) (RR, 0.40; 95% CI, 0.24−0.69), TnI (< 1.23 ng/ml) (RR, 0.45; 95% CI, 0.30−0.67), eGFR (< 48.8 ml/min/1.73 m^2^) (RR, 0.40; 95% CI, 0.24−0.69), or NT-proBNP (< 3,433°pg/ml) (RR, 0.56; 95% CI, 0.41−0.77) values were significantly lower than in studies enrolling patients aged ≥ 70 years (RR, 0.73; 95% CI, 0.48−1.10), with a higher rate of AF (≥ 27.2%) (RR, 0.73; 95% CI, 0.48−1.10) or DM (≥ 38.3%) (RR, 0.69; 95% CI, 0.39−1.21), or with higher LVEF (≥ 37.5%) (RR, 0.75; 95% CI, 0.55−1.00), TnI (≥ 1.23°ng/ml) (RR, 0.81; 95% CI, 0.57−1.15), eGFR (≥ 48.8 mL/min/1.73 m^2^) (RR, 0.74; 95% CI, 0.55−1.00), and NT-proBNP (≥ 3,433°pg/mL) (RR, 0.77; 95% CI, 0.48−1.24) values (Table 5).

**TABLE 5:** Subgroup analyses.

	Subgroups	MACE				HF related rehospitalization			
		Patient’s num.	RR (95% CI)	*P*	*I* ^2^	Patient’s num.	RR (95% CI)	*P*	*I* ^2^
Conditions	AHF	473	0.64 (0.50–0.83)	0.001	87.3	473	0.70 (0.49–0.99)	0.041	85.4
	CHF	693	0.53 (0.39–0.71)	0.000	0	693	0.55 (0.37–0.82)	0.003	36.9
Age (yr)	< 70.0	574	0.54 (0.44–0.67)	0.000	0	574	0.56 (0.40–0.79)	0.001	50.2
	≥ 70.0	592	0.73 (0.48–1.10)	0.133	69.3	592	0.73 (0.48–1.10)	0.133	69.3
AF (%)	< 27.2	595	0.53 (0.43–0.67)	0.000	0	595	0.47 (0.33–0.67)	0.000	0
	≥ 27.2	571	0.72 (0.50–1.05)	0.089	56.6	571	0.93 (0.62–1.41)	0.731	48.1
DM (%)	< 38.3	843	0.61 (0.50–0.76)	0.000	65.7	843	0.61 (0.45–0.82)	0.001	63.5
	≥ 38.3	323	0.48 (0.29–0.79)	0.004	0	323	0.69 (0.39–1.21)	0.194	56.0
Ischemic HF (%)	< 44.2	696	0.61 (0.48–0.76)	0.000	66.5	696	0.71 (0.53–0.96)	0.027	72.3
	≥ 44.2	470	0.55 (0.38–0.79)	0.001	0	470	0.45 (0.26–0.76)	0.003	0
LVEF (%)	< 37.5	470	0.52 (0.37–0.75)	0.000	14.3	470	0.40 (0.24–0.69)	0.001	0
	≥ 37.5	696	0.62 (0.49–0.78)	0.000	62.6	696	0.75 (0.55–1.00)	0.053	66.4
TnI (ng/ml)	< 1.23	451	0.54 (0.42–0.67)	0.000	0.3	451	0.45 (0.30–0.67)	0.000	0
	≥ 1.23	715	0.67 (0.48–0.94)	0.019	57.5	715	0.81 (0.57–1.15)	0.234	59.7
eGFR (ml/min/1.73°m^2^)	< 48.8	470	0.52 (0.37–0.75)	0.000	14.3	470	0.40 (0.24–0.69)	0.001	0
	≥ 48.8	696	0.62 (0.49–0.78)	0.000	62.6	696	0.74 (0.55–1.00)	0.053	66.4
B-lines	< 5.0	493	0.56 (0.42–0.77)	0.000	0	493	0.63 (0.41–0.96)	0.032	49.5
	≥ 5.0	673	0.60 (0.47–0.77)	0.000	67.3	673	0.62 (0.45–0.87)	0.005	67.7
NT-proBNP (pg/ml)	< 3,433	592	0.51 (0.40–0.64)	0.000	0	592	0.56 (0.41–0.77)	0.000	58.4
	≥ 3,433	574	0.76 (0.64–1.07)	0.112	51.7	574	0.77 (0.48–1.24)	0.284	55.6
Follow-up (m)	< 4.7	692	0.66 (0.44–0.98)	0.038	64.4	692	0.65 (0.44–0.97)	0.036	66.5
	≥ 4.7	474	0.56 (0.45–0.69)	0.000	0	474	0.61 (0.43–0.85)	0.004	51.6

### Trial sequential analysis

Assuming a 20% difference between LUS-guided treatment and usual care in the risk of MACEs, TSA showed that the RIS was 2,620 participants. The cumulative Z-curve crossed the trial sequential boundary, indicating a statistically significant difference in the risk of MACEs between the group that underwent LUS-guided treatment and the group that underwent usual care.

In addition, assuming a 20% difference between LUS-guided treatment and usual care in the risk of HF-related rehospitalization, TSA showed that the RIS was 6,437 participants. There was no statistically significant difference in the risk of HF-related rehospitalization between LUS-guided treatment and usual care.

Moreover, assuming a 20% difference between LUS-guided treatment and usual care in the risk of symptomatic HF, TSA showed that the RIS was 4,491 participants. The cumulative Z-curve crossed the trial sequential boundary, indicating a lower risk of MACEs with LUS-guided treatment than with usual care among patients with HF.

## Discussion

This up-to-date meta-analysis of the available evidence showed that LUS can accurately guide the volume management of patients with HF. Not only did LUS not increase the risk of in-hospital adverse events, such as acute kidney injury and hypokalemia, but it shortened the length of hospital stay and reduced the long-term risk of symptomatic HF (RR, 0.37) and readmission for HF (RR, 0.63).

In addition to assessing and monitoring pulmonary congestion, LUS can be used to predict prognosis. In a study involving 150 patients with HF, the Cox regression analysis was used to analyze the relationship between the B-line count at admission and adverse events. The B-line count was significantly correlated with adverse events [hazard ratio (HR), 1.19; *p* = 0.005] (34). Meanwhile, another study showed that bedside LUS could identify patients with acute respiratory distress syndrome who were suitable for and would benefit from prone position ventilation, as well predicting the prognosis of these patients (35). In another study involving patients with acute coronary syndrome, 470 patients underwent LUS within 12 h after admission. The median follow-up period was 5 months (36). The multivariate Cox logistic regression analysis demonstrated that LVEF (HR, 1.45; *p* = 0.040), tricuspid annular systolic displacement (HR, 1.66; *p* = 0.010), and B-line count on LUS (HR, 1.69; *p* = 0.001) were the independent prognostic predictors of MACEs. In another study, a total of 568 patients with acute myocardial infarction (AMI) were divided into the B-line count ≤ 7.5 group (n = 478) and the B-line count > 7.5 group (n = 90). The rates of AHF (62.2 vs. 13.2%), acute kidney injury (26.7 vs. 16.3%), and all-cause mortality (13.3 vs. 3.3%) in the B-line count > 7.5 group were significantly higher than those in the B-line count ≤ 7.5 group (*p* < 0.05) (37), suggesting that LUS can be used to predict the short-term prognosis of patients with AMI.

However, the role of LUS in guiding HF therapy is still controversial. In 2019, the LUS-HF study was published (15). It recruited 123 ambulatory patients with CHF who were followed up for 6 months. The mean LVEF was 39 ± 14%, and the primary endpoint was a composite of urgent visits, hospitalization for worsening HF, and all-cause death. LUS-guided treatment was more effective than usual care in decreasing the primary outcome rate (23 vs. 40%; HR, 0.518; 95% CI, 0.268–0.998), which mainly resulted from the significant decrease in the risk of urgent visits for worsening HF (5 vs. 21%; HR, 0.209; 95% CI, 0.060–0.735). The authors commented on the implications for practice, suggesting that tailored LUS-guided diuretic treatment of pulmonary congestion reduces the number of decompensations and improves walking capacity in patients with HF (15). In another large RCT, a total of 244 patients with CHF and optimized medical therapy were enrolled and randomized into the PE + LUS group (*n* = 127) and the PE only group (*n* = 117). The primary endpoint was the reduction in the rate of hospitalization for acute decompensated heart failure at 90 days. The authors found that the primary endpoint was significantly reduced in the PE + LUS group (9.4 vs. 21.4%; RR, 0.44; 95% CI, 0.23–0.84) (16). However, the BLUSHED-AHF trial showed no clinical benefit of LUS-guided emergency department management for AHF (17). The BLUSHED-AHF trial was a multicenter, single-blind, emergency department-based, pilot trial that randomized 130 patients to undergo 6-h LUS-guided treatment or usual care. Patients were followed up for 90 days after discharge. The primary outcome was a B-line count of ≤ 15 at 6 h. Meanwhile, the number of days alive and out of hospital (DAOOH) at 30 days was the main exploratory outcome. The results demonstrated a similar rate of patients with a B-line count ≤ 15 at 6 h between the LUS-guided treatment group and the usual care group (25.0 vs. 27.5%; *p* = 0.83). In addition, the B-line count at 6 h (35.4 vs. 34.3; *p* = 0.82) and the number of DAOOH (21.3 vs. 21.3 days; *p* = 0.99) were comparable between the two groups. In a subgroup analysis, the authors noted a significantly greater reduction in the B-line count in the LUS-guided treatment group only during the first 48 h (*p* = 0.04).

To date, the most comprehensive meta-analysis included 493 participants from three studies. Our study significantly increases the amount of evidence available for HF-related rehospitalization. In the previous meta-analysis, LUS-guided therapy was associated with a lower rate of urgent care visits (RR, 0.32; 95% CI, 0.18–0.59; *p* = 0.0002) (18). Although there is good consistency between previous RCTs and pooled estimates in this meta-analysis, it is good practice to frame the newest evidence in the context of previous evidence (38). Several recently published RCTs have demonstrated that LUS-guided treatment reduces HF-related rehospitalization in patients with HF compared with usual care (6–10), while more recent systematic reviews, including three with individual patient data, have published meta-analyses contradicting this finding and showing no effect (11–14). Based on these considerations, we believed that it would be helpful to validate the new findings by performing an up-to-date meta-analysis in an attempt to improve the precision of the estimates, reduce variability, and increase generalizability (39). As expected, the addition of the recently published RCT data, which increased the total number of participants, substantially increased the precision of the estimates. The present meta-analysis corroborates the conclusions of all previous meta-analyses, confirming the clinical benefit of LUS-guided treatment for symptomatic HF. However, perhaps unexpectedly, our meta-analysis contradicts recent previous reviews by showing that LUS-guided treatment is also beneficial for HF-related rehospitalization. These two outcomes, along with adverse events, are the only outcomes for which new evidence has been obtained since the most comprehensive meta-analysis to date.

Clinical guidelines for HF management vary in their recommendations on the use of LUS-guided management for patients with HF, which reflects the conflicting evidence in this area of research (20–22). Our study aimed to remove this uncertainty by recruiting more participants to detect a significant difference in the clinically meaningful endpoint of MACEs. Of relevance, the point estimate for MACEs showed LUS-guided treatment to be more effective (RR, 0.59; 95% CI, 0.48–0.71) than was shown in the previous meta-analysis. TSA showed that, assuming a 20% difference between LUS-guided treatment and usual care in the risk of MACEs, the RIS was 2,620 participants, but the accrued information size was 1,203. Therefore, more large-scaled studies are needed for pooled evidence to have sufficient power.

**FIGURE 8:**
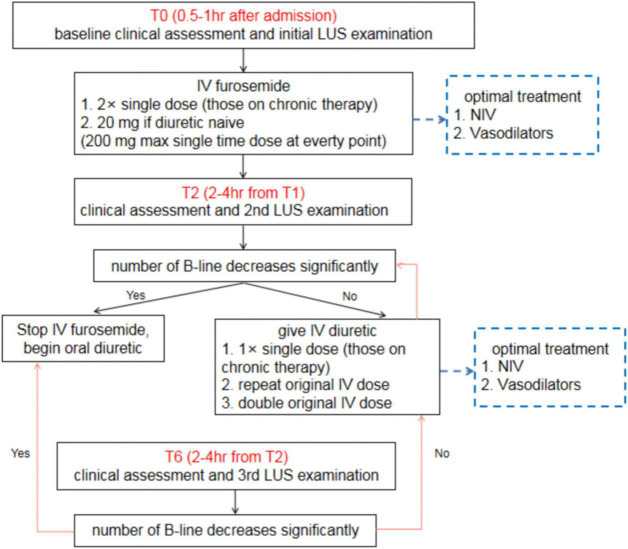
Clinical algorithm for lung ultrasound assessment among patients with acute heart failure. LUS, lung ultrasound; AHF, acute heart failure.

Nevertheless, the evidence for this effect is not yet robust. There is still not sufficient evidence to definitively conclude the effectiveness of LUS-guided treatment in the management of HF, and particularly in more severe HF, such as HF requiring emergency care (40). For patients with AHF, we recommend a simple clinical algorithm involving three time points of LUS assessment (Figure 8). In addition to clinical assessments, all patients with AHF should undergo an immediate baseline LUS assessment at 0.5–1.0 h after admission (T0). A second LUS examination should be performed at 2–4 h after the first assessment (T2), and the final LUS assessment (T6) should be performed at 2–4 h after the second assessment. After the 6-h treatment period, patients should continue to undergo LUS examinations daily throughout hospitalization. For patients with CHF, daily LUS assessment should be performed for up to 7 days or until discharge. The change in B-line count should be used to guide treatment.

In the future studies, the reporting of patient and study characteristics needs to be broader and more detailed to allow further exploration of study populations. Many of the LUS-guided intervention strategies evaluated in this meta-analysis were multifaceted; thus, there is still insufficient clear evidence to suggest clinical benefit with this approach (41). Rather than being a result of simply monitoring B-line counts on LUS, the improved HF outcomes are more likely associated with clinical support, which is indirectly intensified by the use of LUS-guided treatment (42). Therefore, identification of the key components of LUS-guided treatment that are associated with the improvement in HF outcomes is needed.

### Limitations

This study has several limitations that should be noted. First, our meta-analysis is based on study-level data, and it is possible that there are flaws in the original studies. Second, two studies are still in the stage of subject enrollment; therefore, the overall outcome may be affected by the results of these studies. Third, there is a risk of geographical variation. The 10 studies showed slight differences in patients’ characteristics, conditions, LUS-guided treatment strategies, and follow-up periods. Fourth, the timing of LUS may have affected the results. In a study involving 60 HF inpatients, a B-line count ≥ 30 at discharge was a strong outcome predictor (all-cause death or HF hospitalization) (HR, 5.66) (43). This finding was confirmed in another study, which indicated that a B-line count > 15 before discharge (HR, 11.74) was an independent predictor of rehospitalization at 6 months among 100 patients with AHF (13). In another study involving 162 HF patients, although both the B-line counts at admission and discharge were positively correlated with BNP concentration and clinical congestion score (*p* < 0.05), only the B-line count at discharge predicted the risk of re-hospitalization for HF or death at 6 months (HR, 1.16) (44). In our study, the meta-regression analysis showed a significant correlation among MACEs, HF rehospitalization, symptomatic HF, and change in B-line count (*p* < 0.05). However, LUS was performed at admission in most studies. Therefore, multiple LUS examinations may improve the clinical outcomes of patients. Fifth, the sample size of this meta-analysis was small. TSA showed that, assuming a 20% difference in the risk of MACEs between LUS-guided treatment and usual care, the RIS was 2,620 participants; however, the accrued information size was 1,203. Finally, the median follow-up period was 4.7 months. However, HF outcomes are expected to increase over time; thus, studies with longer follow-up periods are needed.

## Conclusion

The current pooled evidence indicates a beneficial effect of LUS-guided therapy on MACEs, HF-related rehospitalization, and symptomatic HF compared with usual care. However, the sample size was still small, and there is insufficient evidence to reach a definitive conclusion on the effectiveness of LUS-guided treatment in the management of HF.

## Data availability statement

The raw data supporting the conclusions of this article will be made available from the corresponding author by request.

## Author contributions

YL wrote the main manuscript text. HA, NM, and PL analyzed the data. JR designed the study. All authors contributed to the article and approved the submitted version.

## Footnotes

1
www.ctu.dk/tsa
